# Methamphetamine and Methamphetamine-Induced Neuronal Exosomes Modulate the Activity of Rab7a via PTEN to Exert an Influence on the Disordered Autophagic Flux Induced in Neurons

**DOI:** 10.3390/ijms26062644

**Published:** 2025-03-14

**Authors:** Hai Qiu, Manting Zhang, Minchun Li, Chuanxiang Chen, Huijun Wang, Xia Yue

**Affiliations:** 1 School of Forensic Medicine, Southern Medical University, Guangzhou 510515, China; tzonerqiuhai@i.smu.edu.cn (H.Q.); zmt1996@i.smu.edu.cn (M.Z.); chanel00@smu.edu.cn (C.C.); 2 Guangzhou Key Laboratory of Forensic Multi-Omics for Precision Identification, School of Forensic Medicine, Southern Medical University, Guangzhou 510515, China; 3 Department of Medical Genetics, School of Basic Medical Sciences, Southern Medical University, Guangzhou 510515, China; lmc1997@smu.edu.cn

**Keywords:** methamphetamine, neuron injury, autophagic flux, Rab7a, PTEN, exosomes, vesicular transport

## Abstract

Autophagy is a critical mechanism by which methamphetamine (METH) induces neuronal damage and neurotoxicity. Prolonged METH exposure can result in the accumulation of autophagosomes within cells. The autophagy process encompasses several essential vesicle-related biological steps, collectively referred to as the autophagic flux. However, the precise mechanisms by which METH modulates the autophagic flux and the underlying pathways remain to be elucidated. In this study, we utilized a chronic METH exposure mouse model and cell model to demonstrate that METH treatment leads to an increase in p62 and LC3B-II and the accumulation of autophagosomes in striatal neurons and SH-SY5Y cells. To assess autophagic flux, this study utilized autophagy inhibitors and inducers. The results demonstrated that the lysosomal inhibitor chloroquine exacerbated autophagosome accumulation; however, blocking autophagosome formation with 3-methyladenine did not prevent METH-induced autophagosome accumulation. Compared to the autophagy activator rapamycin, METH significantly reduced autophagosome–lysosome fusion, leading to autophagosome accumulation. Rab7a is a critical regulator of autophagosome–lysosome fusion. Although Rab7a expression was upregulated in SH-SY5Y cells and brain tissues after METH treatment, immunoprecipitation experiments revealed weakened interactions between Rab7a and the lysosomal protein RILP. Overexpression of active Rab7a (Rab7a Q67L) significantly alleviated the METH-induced upregulation of LC3-II and p62. PTEN, a key regulator of Rab7a dephosphorylation, was downregulated following METH treatment, resulting in decreased Rab7a dephosphorylation and reduced Rab7a activity, thereby contributing to autophagosome accumulation. We further investigated the role of neuronal exosomes in the autophagy process. Our results demonstrated that the miRNA expression profiles in exosomes released by METH-induced SH-SY5Y cells were significantly altered, with 122 miRNAs upregulated and 151 miRNAs downregulated. KEGG and GO enrichment analyses of these differentially expressed miRNAs and their target genes revealed significant associations with the autophagy pathway and potential regulation of PTEN expression. Our experiments confirmed that METH-induced exosomes reduced PTEN expression levels and decreased Rab7a dephosphorylation, thereby exacerbating autophagic flux impairment and autophagosome accumulation. In conclusion, our study indicated that METH and its induced neuronal exosomes downregulate PTEN expression, leading to reduced Rab7a dephosphorylation. This, in turn, hinders the fusion of autophagosomes and lysosomes, ultimately resulting in autophagic flux impairment and neuronal damage.

## 1. Introduction

Methamphetamine (METH) is a widely abused amphetamine-type psychostimulant characterized by its high potential for addiction and significant neurotoxic properties, leading to a range of neuropsychiatric consequences (e.g., neurodegenerative diseases like Alzheimer’s disease and Parkinson’s disease and psychosis like agitation, anxiety, hallucinations, and paranoia), sharing similar symptoms or mechanisms [1]. The molecular mechanisms underlying these neuropsychiatric conditions resulting from chronic METH abuse currently and primarily involve dopaminergic and serotonergic disfunction, neuronal injury, oxidative stress, neuroinflammation, and associated factors [2].

A series of cellular stress events induced by methamphetamine (METH) rapidly initiates autophagy. Our previous studies have demonstrated alterations in autophagy following METH exposure [3,4,5,6]. Autophagy, a highly conserved and strictly regulated catabolic pathway across species, plays a pivotal role in the degradation of aberrant proteins and the maintenance of cellular energy homeostasis in neurons. In 2002, Kristin E Larsen et al. discovered autophagosomes in the cytoplasm of primary midbrain neurons exposed to METH using electron microscopy [7]. Since then, the role of autophagy in METH-induced neurotoxicity has been extensively studied. However, the precise effects of METH on autophagy remain uncertain. Lin et al. showed that the autophagy mechanism is a protective factor against METH neurotoxicity [8]. Conversely, Li and Xu et al. considered that METH exposure upgrades autophagy markers, which triggers neuronal apoptosis [5,6]. Chutikorn et al. conducted in vitro experiments on SK-n-SH dopaminergic cell lines and revealed that METH enhances autophagy by inhibiting the dissociation of the Bcl-2/Beclin 1 complex and the associated upstream pathway, ultimately leading to cell death [9]. Similarly, in vivo studies on rats indicate that METH exposure triggers early autophagy, which progressively proceeds to apoptosis during the early pathophysiological process in striatum [2,10]. However, Ryskalin et al. [11] and Xie et al. [12] argued that METH exposure induces neurotoxicity by weakening autophagy. Several treatment regimens or therapeutic agents, such as Curcumin [11] and Lactulose [12], have been shown to ameliorate METH-induced neurotoxic damage and exert their cytoprotective effects by alleviating METH-induced autophagy inhibition. Therefore, it seemingly becomes imperative to elucidate the alterations in the autophagy pathway following METH exposure.

Assessing autophagic flux represents a critical methodology for evaluating the impact of drugs on autophagy. Autophagic flux is a dynamic, multi-step process that occurs over a defined time period. This process involves the activation of autophagy-related proteins, cargo selection and sequestration within autophagosomes, fusion of autophagosomes with lysosomes, subsequent degradation, and the release of resulting macromolecules back into the cytosol [13]. Similar to other cellular pathways, autophagic flux can be modulated at various stages following METH exposure. However, little is known about how METH affects autophagy flow.

Rab7a is an indispensable protein in vesicular transport processes involving autophagic flux, and it is the most important subtype of Rab7 protein involved in autophagy and mitophagy [14,15]. As the sole lysosomal Rab protein within the Rab GTPase superfamily, Rab7a regulates trafficking from late endosomes to lysosomes and plays a unique role in autophagy, particularly in promoting autophagosome–lysosome fusion [16]. As a GTPase, the activation state of Rab7a depends on its binding to either GTP or GDP. The transition between the inactive (GDP-bound) and active (GTP-bound) states of Rab7a governs its interaction with effector proteins and influences their biological functions [17]. Inactivation of Rab7a results in the accumulation of autophagosomes, underscoring the importance of activated Rab7a in ensuring the efficiency of autophagosome degradation [18]. The activity and function of Rab7a are intricately linked to various post-translational modifications, including phosphorylation [19], succinylation [20], palmitoylation [21], and C-terminal prenylation (which is essential for its membrane insertion [22]). Specifically, dephosphorylation at the S72 residue of Rab7a plays a critical role in facilitating the delivery of Rab7 to endosomal membranes, a process that is closely associated with GTP binding [23]. This dephosphorylation event can be modulated by the dual-specificity phosphatase PTEN, which promotes Rab7 dephosphorylation and regulates its localization to late endosomal membranes, thereby contributing to endosomal maturation [19].

Exosomes, which play a crucial role in neuronal signaling, may also influence PTEN expression levels in neurons. Exosomes are small vesicles actively secreted by various living cells, typically ranging from 40 to 200 nm in diameter. They possess a lipid bilayer membrane structure, exhibit stability, and contain a diverse array of biomolecules that reflect the cellular state and participate in pathophysiological processes [24]. Exosomes from neurons, astrocytes, oligodendrocytes, and microglia facilitate intracellular communication by transferring proteins and genomic materials. This widespread and extremely important process can support normal neuronal function but may also spread pathological proteins or cause cytopathological changes to healthy neurons in neurodegenerative diseases [25,26]. Exosomes, derived from a variety of cells within and outside the central nervous system, exert significant biological effects on neurons. They have emerged as a focal point in the investigation of diagnosis, pathological mechanisms, and therapeutic approaches in neurodegenerative diseases and neural regeneration, demonstrating considerable potential [27]. The characteristic proteins and miRNAs associated with neurodegenerative diseases found in exosomes have been validated as diagnostic markers and signaling molecule [28,29,30]. METH-induced alterations in neuronal exosomal miRNA profiles may play a significant role in METH-induced neurotoxicity; however, this area remains underexplored. Additionally, the impact of METH-induced changes in neuronal exosomes on autophagy has not yet been reported.

The objective of this study is to elucidate the specific impacts of methamphetamine on each phase of neuronal autophagy, the role of neuronal exosomes in modulating autophagy, and the associated neurotoxic effects.

## 2. Results

### 2.1. Exposure to Methamphetamine Induces Autophagosome Accumulation Both In Vivo and In Vitro

**Figure 1:**
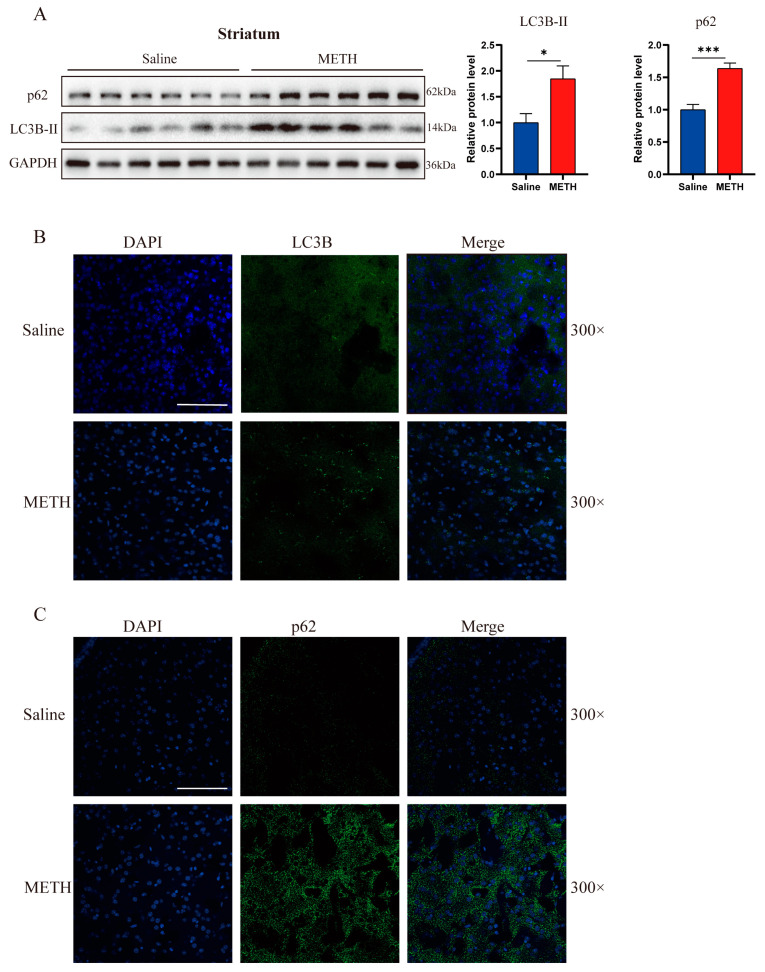
Accumulation of autophagosomes was observed in a mouse model of chronic METH intoxication; the expression levels of LC3B and p62 in the striatum were evaluated. (**A**) Western blot analysis and quantitative assessments were conducted to evaluate the expression of LC3B and p62 in the striatum following METH exposure (*n* = 6 mice/group). GAPDH served as an internal control. The bars in the right histogram represent the mean ± SEM of the corresponding protein levels. Compared to the control group, statistical differences are indicated by * *p* < 0.05 and *** *p* < 0.001. (**B**) Representative images of immunofluorescence staining of LC3B (green) and DAPI (blue). (**C**) Representative images of immunofluorescence staining of p62 (green) and DAPI (blue). The images of B and C were captured at a magnification of 400× and subsequently downscaled by a factor of 1.5. Scale bars represent 100 μm.

To investigate the impact of METH exposure on autophagy, we evaluated the expression levels of LC3B-II and p62 in striatal neurons of METH-treated mice using Western blot and immunofluorescence. Western blot analysis revealed a significant increase in LC3B-II and p62 expression compared to the saline control group (Figure 1A). Immunofluorescence staining demonstrated a notable increase in LC3B- and p62-positive puncta in the striatal neurons of METH-treated mice (Figure 1B).

**Figure 2:**
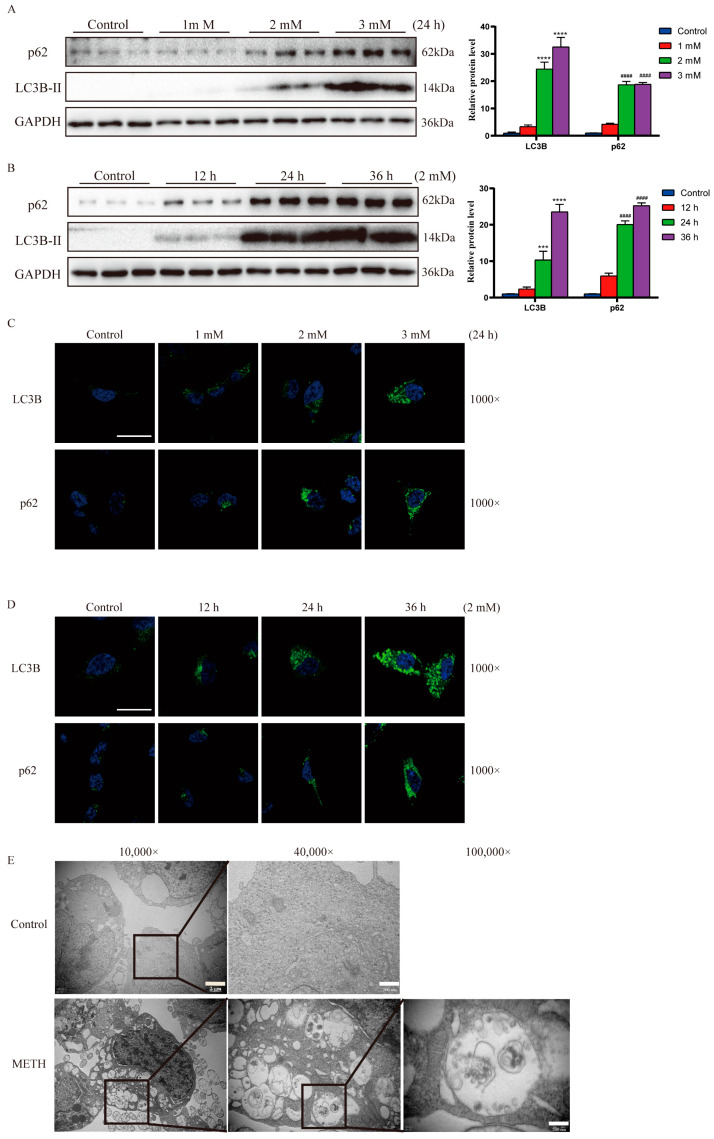
TExposure to METH results in the accumulation of autophagosomes in vitro. (**A**,**B**) Western blot analysis and quantitative assessments were conducted to evaluate the expression levels of LC3B and p62 following METH exposure in SH-SY5Y cells (*n* = 3 of cell passages/group). GAPDH served as an internal control. The bars in the right histogram represent the mean ± SEM of the corresponding protein levels. Compared to the control group, statistical differences are indicated by *** *p* < 0.001 and **** *p* < 0.0001 for LC3B, and #### *p* < 0.0001 for p62. (**C**,**D**) Representative images of immunofluorescence staining of LC3B and p62. The images were captured under a magnification of 1000×. Scale bars represent 20 μm. (**E**) The ultrastructure of autophagosomes in SH-SY5Y cells following METH exposure was examined using transmission electron microscopy. The images were captured under a magnification of 10,000×, 40,000×, and 100,000×. Scale bars represent 2 μm, 500 nm, and 200 nm.

Additionally, SH-SY5Y cells were exposed to varying concentrations of METH (0, 1.0, 2.0, and 3.0 mM) for different durations (0, 12, 24, or 36 h), and the expression of LC3B-II and p62 was assessed using Western blot and immunofluorescence. The results exhibited a significant dose- and time-dependent increase in LC3B-II and p62 expression in METH-treated SH-SY5Y cells (Figure 2A–D). To more effectively visualize the accumulation of autophagosomes, transmission electron microscopy (TEM), considered the gold standard for autophagy identification [31], was employed. TEM analysis revealed an increased presence of double-membrane structures in METH-treated SH-SY5Y cells compared to the control group (Figure 2E).

Autophagosome accumulation can be attributed to three main factors: (i) increased autophagosome formation, (ii) decreased autophagosome degradation, or (iii) a combination of both. To elucidate the potential mechanisms underlying METH-induced autophagosome accumulation, the integrity of the autophagy machinery was assessed at various levels.

### 2.2. Methamphetamine Exposure Induces an Upregulation of Autophagosome Formation

**Figure 3:**
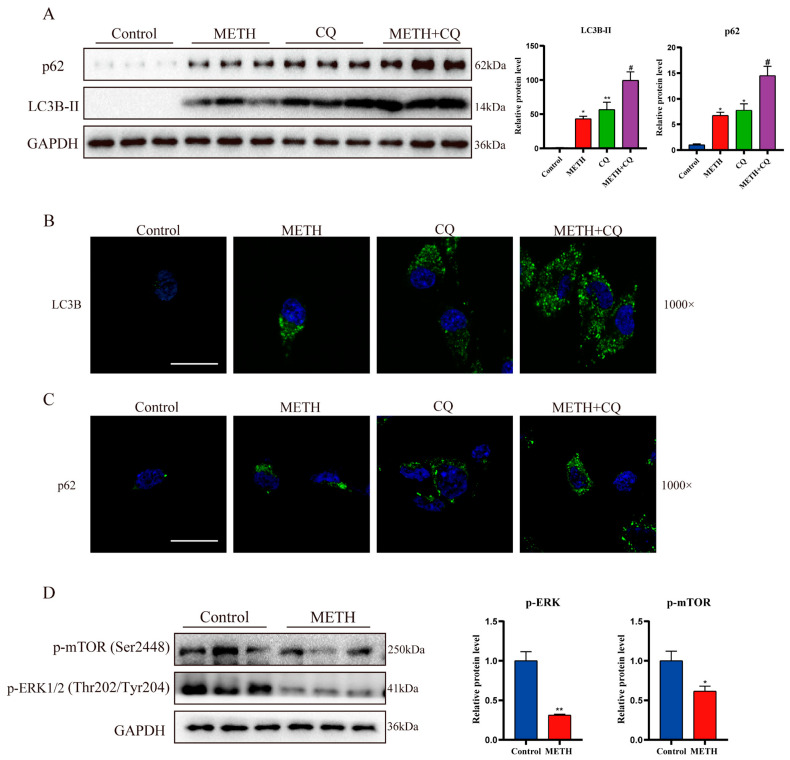
METH induces increased autophagosome synthesis in vitro. SH-SY5Y cells were pretreated with chloroquine (CQ, 200 nM, an inhibitor of lysosomal degradation of autophagosomes) for 1 h prior to METH (2.0 mM) exposure for 24 h. (**A**) Western blot analysis was used to detect autophagy markers in the presence of CQ and METH (*n* = 3 of cell passages/group). The results for LC3B-II and p62 protein expression are presented in the right histogram. GAPDH served as an internal control. Compared to the control group, statistical differences are indicated by * *p* < 0.05 and ** *p* < 0.01. Compared to the METH group, statistical differences are indicated by # *p* < 0.05 (*n* = 3 of cell passages/group). (**B**,**C**) Immunofluorescence staining was used to detect LC3B and p62 puncta formation in the cytoplasm following CQ inhibition. The images were captured under a magnification of 1000×. Scale bars represent 20 μm. (**D**) Western blot analysis was conducted to evaluate the expression levels of p-ERK1/2 (Thr202/Tyr204) and p-mTOR (Ser2448) (*n* = 3 of cell passages/group). The Western blot analysis results of p-ERK1/2 (Thr202/Tyr204) and p-mTOR (Ser2448) proteins are presented in the right histogram. GAPDH served as an internal control. Compared to the control group, statistical differences are indicated by * *p* < 0.05 and ** *p* < 0.01.

To investigate the impact of METH exposure on autophagosome generation in vitro, we employed chloroquine (CQ), a pharmacological inhibitor of lysosomal degradation, to impede autophagic flux. Treatment with CQ resulted in the accumulation of LC3B-II and p62 in METH-treated SH-SY5Y cells (Figure 3A). Subsequently, immunofluorescence analysis revealed an increased abundance of LC3B- and p62-positive puncta in METH-exposed SH-SY5Y cells in the presence of CQ and METH (Figure 3B,C). To elucidate the pathway by which METH triggers autophagosome generation, we examined the upstream regulators of autophagy. The results showed that the levels of p-ERK1/2 (Thr202/Tyr204) and p-mTOR (Ser2448) were decreased after METH exposure, suggesting that METH may induce autophagosome generation via the ERK/mTOR pathway (Figure 3D).

**Figure 4:**
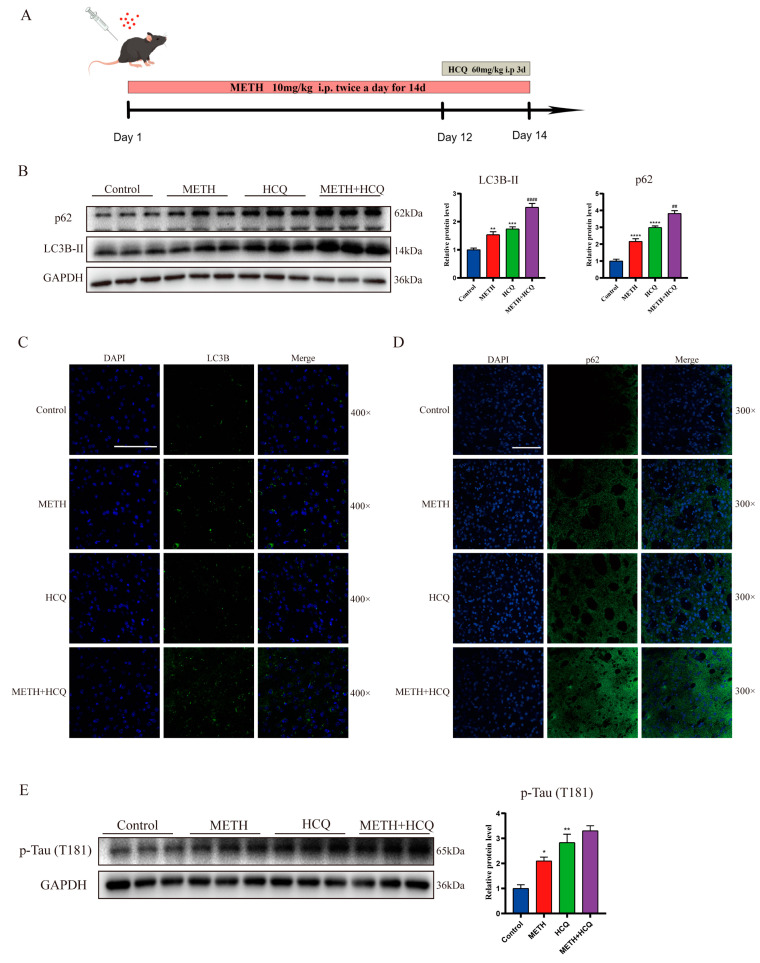
METH resulted in decreased autophagy flux in vivo. (**A**) Schematic diagram of the administration process in mice. (**B**) Western blot analysis and quantitative assessments were conducted to evaluate the expression levels of LC3B and p62 following METH exposure in the striatum (*n* = 6 mice/group). GAPDH served as an internal control. Compared to the control group, statistical differences are indicated by ** *p* < 0.01, *** *p* < 0.001, and **** *p* < 0.0001. Compared to the METH group, statistical differences are indicated by ## *p* < 0.01 and #### *p* < 0.0001. (**C**) Representative images of immunofluorescence staining of LC3B (green) and DAPI (blue). The images were captured under a magnification of 400×. Scale bars represent 100 μm. (**D**) Representative images of immunofluorescence staining of p62 (green) and DAPI (blue). The images were captured under a magnification of 400× and subsequently downscaled by a factor of 1.5. Scale bars represent 100 μm. (**E**) Western blot analysis was conducted to evaluate p-Tau protein expression in the striatal (*n* = 3 mice/group). The results for p-Tau protein expression are presented in the right histogram. GAPDH served as an internal control. Compared to the control group, statistical differences are indicated by * *p* < 0.05 and ** *p* < 0.01.

To verify autophagic flux under in vivo conditions, we administered daily injections of 60 mg/kg hydroxychloroquine (HCQ), a derivative of CQ, to mice during the final three days of chronic METH intoxication modeling (Figure 4A). Consistent with our in vitro findings, METH treatment significantly increased the accumulation of LC3B-II and p62, as detected by Western blot and immunofluorescence in the presence of HCQ and METH (Figure 4B,C). These results collectively indicate that methamphetamine exposure leads to increased autophagosome generation both in vitro and in vivo.

Furthermore, we assessed p-Tau protein levels in a chronic METH poisoning mouse model. Through Western blot analysis (Figure 4E), we observed a notable increase in p-Tau protein accumulation within the striatum region of both METH-intoxicated mice and those treated with HCQ, compared to the control group. This finding suggests that the inhibition of autophagic flux in neuronal cells leads to cytoplasmic accumulation of p-Tau protein.

### 2.3. Methamphetamine Exposure Leads to a Decrease in Autophagosome Degradation In Vitro

**Figure 5:**
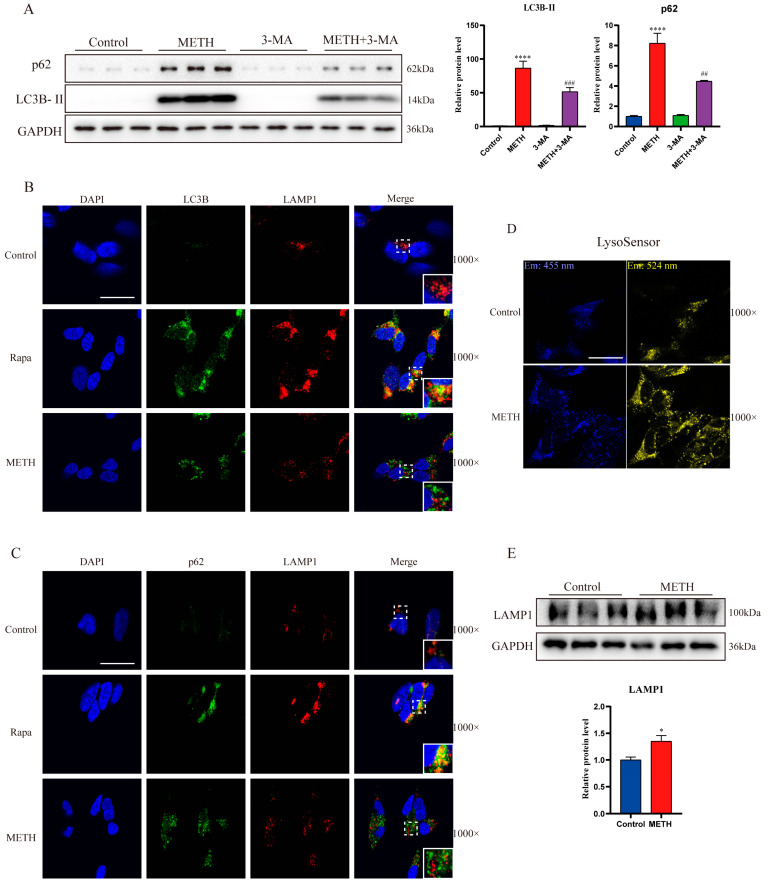
In vitro experiments demonstrated that METH impaired autophagosome degradation. SH-SY5Y cells were pretreated with 3-MA (5.0 mM) for 1 h prior to METH (2.0 mM) exposure for 24 h. (**A**) Western blot analysis was conducted to evaluate autophagy markers in the presence of 3-MA and METH. The results for LC3 and p62 protein expression are presented on the right. GAPDH served as an internal control. Compared to the control group, statistical differences are indicated by **** *p* < 0.0001. Compared to the METH group, statistical differences are indicated by: ## *p* < 0.01 and ### *p* < 0.001. (*n* = 3 of cell passages/group). (**B**,**C**) Immunofluorescence microscopy was used to detect the colocalization of autophagy markers (green) and lysosomal markers (red) following METH and autophagy inducer Rapa treatment, respectively. The images were captured under a magnification of 1000×. Scale bar represents 20 μm. (**D**) LysoSensor probe method was employed to detect the pH value of lysosomes. Scale bar represents 20 μm. (**E**) Western blot analysis was conducted to evaluate the expression of the lysosomal marker LAMP1. The results for LAMP1 protein expression are presented below. GAPDH served as an internal control. Compared to the control group, statistical differences are indicated by * *p* < 0.05.

To investigate whether METH exposure hinders the autophagosome degradation process, we utilized 3-MA to inhibit autophagosome formation and nucleation prior to METH exposure in SH-SY5Y cells. As shown in Figure 5A, even after 3-MA effectively blocked autophagosome generation, METH exposure still resulted in increased accumulation of LC3B and p62. To elucidate the mechanism underlying METH-induced impairment of autophagic degradation, we examined the colocalization of autophagosome markers and lysosome markers.

In untreated physiological condition cells, intracellular LC3B predominantly exists as LC3B-II, making it challenging to observe. To visualize normal autophagy flux, we employed rapamycin (RAPA), an autophagy activator, as a positive control. As shown in Figure 5B,C, compared with the fusion of autophagosomes (green fluorescence) and lysosomes (red fluorescence) into autophagolysosomes (yellow puncta) after RAPA treatment, fewer yellow puncta were observed following METH treatment, indicating that METH exposure impairs the fusion of autophagosomes and lysosomes.

Furthermore, we assessed alterations in lysosomal pH using LysoSensor Yellow/Blue DND-160 and quantified lysosome marker levels via Western blot analysis in SH-SY5Y cells. As shown in Figure 5D,E, METH exposure led to a decrease in lysosomal pH value (more acidic) (Figure 5D) or an increase in LAMP1 (lysosome marker, Figure 5E). These findings suggest that METH inhibits autophagosome degradation by impeding the transport and fusion processes of autophagy rather than by directly inhibiting lysosomal function.

### 2.4. METH Inhibits the Transport and Fusion of Autophagosome by Inhibiting Rab7a Activity

As previously discussed, METH inhibits post-autophagosomal transport and fusion with functional endolysosomal compartments. In mammalian cells, Rab7a is predominantly localized in late endosomes and plays a critical role in regulating the transport of these vesicles to functional endolysosomal compartments [32]. Modulation of Rab7a activity is a key regulatory event in autophagosome transport and fusion [16,17,33]. METH potentially disrupts autophagic flux by influencing Rab7a activity. To test this hypothesis, we conducted the following experiments.

**Figure 6:**
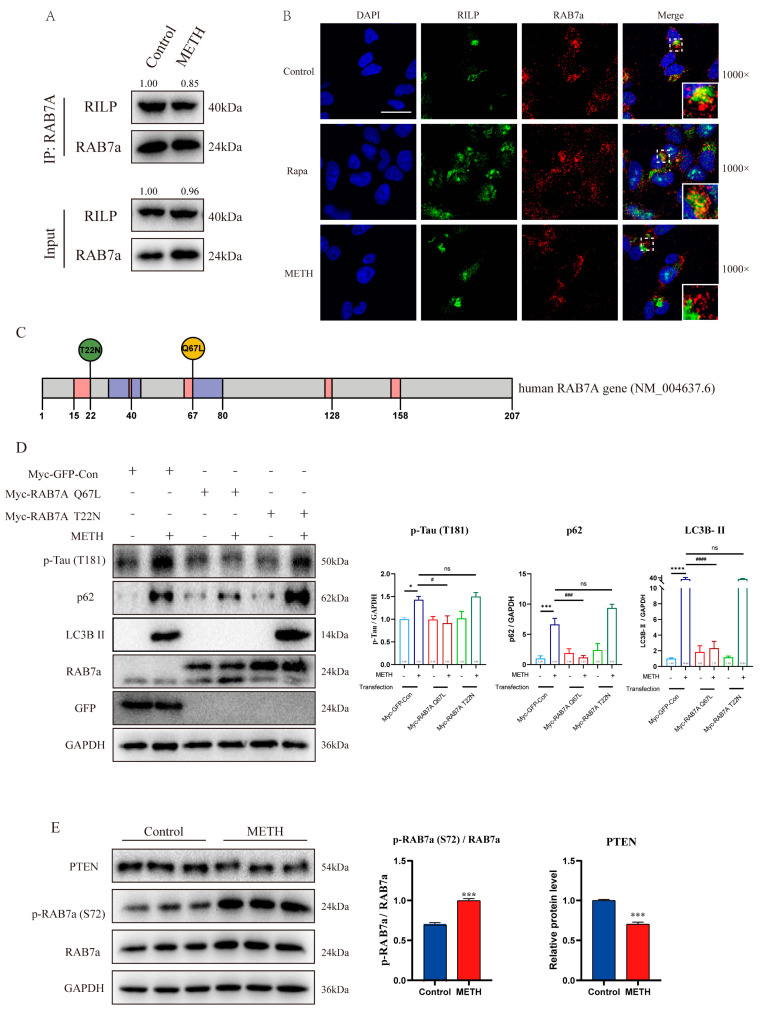
METH inhibits the transport and fusion of autophagosomes by suppressing Rab7a activity. (**A**) CO-IP results showing the interaction between RAB7a and RILP. The ratio of RILP to RAB7a is indicated above. (**B**) Immunofluorescence images of SH-SY5Y cells demonstrating colocalization (yellow) of RILP protein (green) and RAB7a (red) following treatment with Rapa (500 nM, 24 h) and METH (2.0 mM, 24 h). The images were captured under a magnification of 1000×. Scale bar represents 20 μm. (**C**) Constructs of the dominant active mutant (RAB7A Q67L, yellow site) and dominant inactive mutant (RAB7A T22N, green site) of the RAB7A gene were generated. (**D**) Western blot analysis was conducted to evaluate LC3B-II, p62 and autophagy substrate (p-Tau) after RAB7A overexpression in SH-SY5Y cells (*n* = 3 of cell passages/group). The results for p-Tau, p62 and LC3B-II protein expression are presented in the right histogram. GAPDH served as an internal control. In the statistical comparison of data corresponding to the two endpoints of the connection, symbols * and # are used to denote significant differences. The statistical significances are shown as: * *p* < 0.05, *** *p* < 0.001, **** *p* < 0.0001; ns *p* > 0.05, # *p* < 0.05, ### *p* < 0.001, #### *p* < 0.0001. (**E**) Western blot analysis was conducted to evaluate p-Rab7a and PTEN after 2.0 mM METH exposure in SH-SY5Y cells (*n* = 3 of cell passages/group). The results for p-RAB7a/RAB7a and PTEN protein expression are presented in the right histogram. GAPDH served as an internal control. Compared to the control group, statistical differences are indicated by *** *p* < 0.001.

The activation state of Rab7a can be assessed by examining its interaction with Rab7a-interacting lysosomal protein (RILP), a significant downstream effector involved in autophagosome–lysosome fusion [34,35,36]. Co-Immunoprecipitation (CO-IP) experiments were performed to define the kinetics of Rab7a-RILP association, revealing that METH exposure reduced the amount of RILP co-precipitated with Rab7a (Figure 6A). To further corroborate these findings, immunofluorescence was used to observe the co-localization of Rab7a and RILP. Green fluorescently labeled RILP and red fluorescently labeled Rab7a formed yellow plaques after rapamycin treatment, while fewer yellow plaques were observed in the METH group, indicating weakened co-localization between RILP and Rab7a (Figure 6B). These co-localization results from CO-IP and immunofluorescence suggest that METH impairs the effective binding between Rab7a and RILP, thereby affecting Rab7a activity.

To further investigate the relationship between METH-induced inhibition of autophagic flux and Rab7a activation, constitutively active (Rab7a Q67L) and dominant-negative (Rab7a T22N) mutants were constructed and transfected into SH-SY5Y cells (Figure 6C). Compared to the GFP control and inactive Rab7a (Rab7a T22N) overexpression groups, overexpression of active Rab7a (Rab7a Q67L) significantly alleviated METH-induced upregulation of LC3-II and p62, as well as the accumulation of the autophagy substrate p-Tau (Figure 6D).

To explore how METH reduces Rab7a activity, we examined the dephosphorylation of Rab7a, which is essential for its activation. Following METH exposure, Rab7a phosphorylation was significantly increased, while PTEN, an enzyme responsible for Rab7a dephosphorylation [24], was reduced (Figure 6E). These findings suggest that METH diminishes Rab7a dephosphorylation by inhibiting PTEN, thereby decreasing Rab7a activity.

### 2.5. METH Can Induce Neurons to Release Exosomes Containing Differential Express miRNAs (DE-miRNAs) and Lead to a Decrease in the Level of PTEN in Neurons, Thereby Affecting the Phosphorylation Level of Rab7a

**Figure 7:**
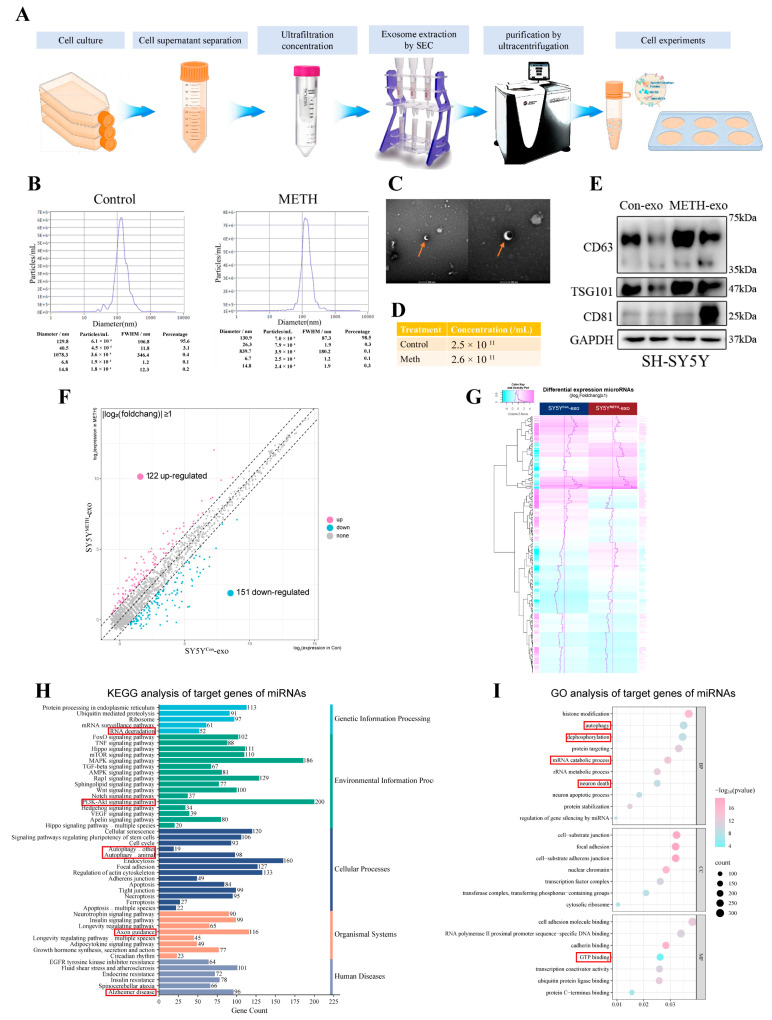
Characterization of exosomes derived from METH-induced SH-SY5Y cells and bioinformatics analysis of differentially expressed miRNAs. (**A**) Schematic illustration of the workflow for exosome extraction and application. (**B**) NTA identification of exosomes. (**C**) Electron microscopic identification of exosomes. (**D**) Nanoparticle counting of exosomes by NTA. (**E**) Western blot analysis to confirm the presence of exosomal markers CD63, TSG101, and CD81. (**F**,**G**) Symmetrical scatter plots and heat maps of the expression differences of exosomal miRNAs. (**H**,**I**) KEGG and GO analyses of the predicted target genes of DE-miRNAs in exosomes. The red box highlights the KEGG pathway and GO entries related to METH-induced autophagy in neurons.

To investigate the role of neuronal exosomes in METH-induced autophagic flux impairment, we utilized SH-SY5Y cells as a model to examine the expression profiles of miRNAs in exosomes secreted by these cells following exposure to 2.0 mM METH. We carried out the extraction of exosomes in accordance with the flowchart (Figure 7A). NTA results demonstrated that over 95% of nanoparticles in both the control and METH-treated groups exhibited a diameter of approximately 130 nm, consistent with the established size range for exosomes (Figure 7B). Subsequently, TEM revealed that the extracted exosomes displayed the characteristic “cup-shaped” morphology typical of exosomes (Figure 7C). Finally, we detected the expression levels of the exosomal marker proteins CD81, CD63, and TSG101 using Western blot analysis (Figure 7E). These findings confirmed that the extracted exosomes exhibited typical exosomal characteristics. Furthermore, we conducted exosome characterization using NTA, which indicated an exosome concentration of 2.5 × 10^11^ particles/mL in the control group and 2.6 × 10^11^ particles/mL in the METH-treated group (Figure 7D).

**Figure 8:**
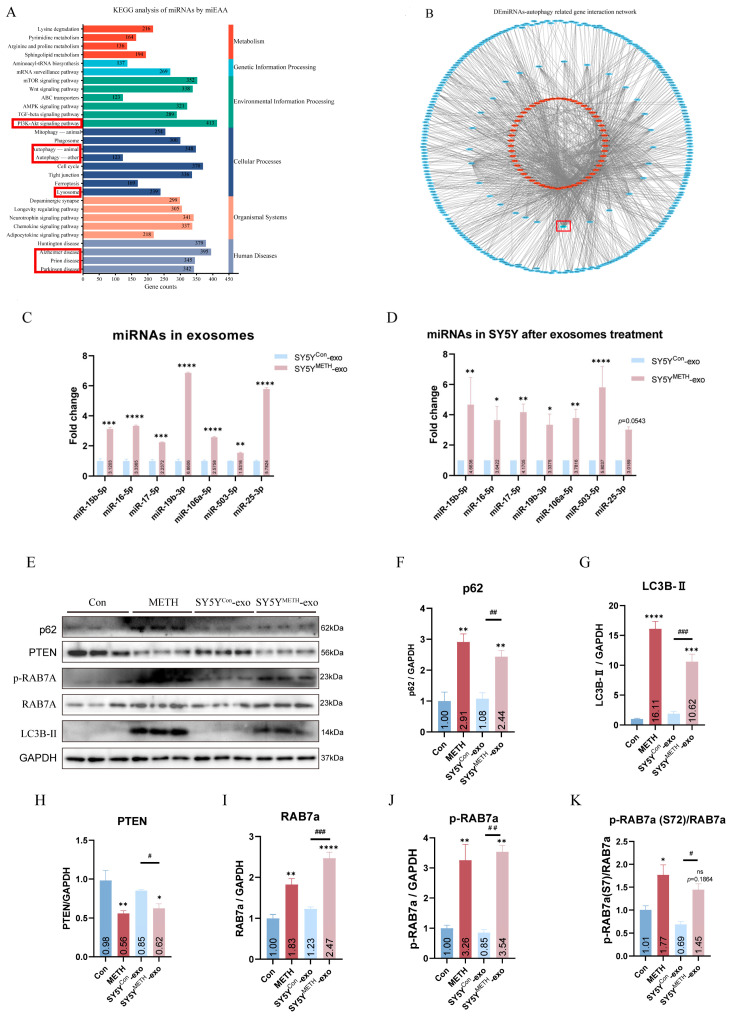
Following the exposure of SH-SY5Y cells to exosomes released from METH-induced SY5Y cells, the miRNA molecular profile was altered, subsequently impacting the PTEN/RAB7A signaling pathway and exacerbating autophagic flux impairment. (**A**) KEGG pathway enrichment of DEmiRNAs by miEAA. The red box highlights the KEGG pathway entries related to METH-induced autophagy in neurons. (**B**) The interaction network of DE-miRNAs and autophagy-related genes. The red box indicates that PTEN is a common target gene for multiple DEmiRNAs. (**C**) qPCR verification of selected DE-miRNAs in exosomes. The statistical significance is shown as: ** *p* < 0.01, *** *p* < 0.001, **** *p* < 0.0001. (**D**) qPCR verification of selected DE-miRNAs in exosome-treated SY5Y. The statistical significance is shown as: * *p* < 0.05, ** *p* < 0.01, **** *p* < 0.0001. (**E**–**K**) Western blot analysis of p62, PTEN, LC3B-II, RAB7a and p-RAB7a (S72). Compare with the Control group, the significant differences above the bars are shown as: ns *p* > 0.05, * *p* < 0.05, ** *p* < 0.01, **** *p* < 0.0001. While in the statistical comparison of data corresponding to the two endpoints of the connection, symbols # is used to denote significant differences, which are shown as: # *p* < 0.05, ## *p* < 0.01, ### *p* < 0.001.

Through miRNA microarray analysis, we identified 273 significantly DE-miRNAs (fold change ≥ 2 or ≤0.5) in the exosomes from the control and METH-treated groups. Among these, 122 miRNAs were upregulated and 151 were downregulated (Figure 7F,G). Subsequently, we utilized the miRTarBase database to predict target genes for differentially expressed miRNAs (DE-miRNAs) and identified genes with robust miRNA-target gene interaction relationships as potential targets. These target genes were subjected to KEGG pathway enrichment analysis and GO functional enrichment analysis. KEGG enrichment analysis revealed that pathways related to autophagy, RNA degradation, the PI3K-AKT signaling pathway, axon guidance, and Alzheimer’s disease were significantly enriched (Figure 7H). The results of GO enrichment analysis indicate that the biological processes (BP) associated with differentially expressed target genes are primarily involved in autophagy, dephosphorylation, mRNA metabolic processes, and neuron death. Additionally, the molecular functions (MF) are predominantly related to GTP binding (Figure 7I). miEAA was employed to predict the KEGG pathway enrichment of DE-miRNAs. The results were consistent with those of the target gene enrichment analysis, demonstrating significant enrichment in pathways related to autophagy, lysosome function, the PI3K-AKT signaling pathway, and Alzheimer’s disease (Figure 8A). We constructed an interaction network between DE-miRNAs and autophagy-related target genes, revealing that PTEN can be regulated by multiple DE-miRNAs (Figure 8B).

We validated the expression of several differentially expressed miRNAs (DE-miRNAs) in exosomes, including miR-15b-5p, miR-16-5p, miR-17-5p, miR-19-3p, miR-106-5p, miR-503-5p, and miR-25-3p. The observed increase in their expression levels was consistent with the sequencing data (Figure 8C). Furthermore, we confirmed that the expression levels of these DE-miRNAs were significantly elevated in SH-SY5Y cells following exosome treatment (Figure 8D). Subsequently, we examined the protein expression levels in SH-SY5Y cells following exosome treatment (Figure 8E). The results demonstrated that PTEN expression was reduced and RAB7A and p-RAB7A (S72) were downregulated, while the ratio of p-RAB7A to RAB7A increased (Figure 8H–K). Concurrently, the expressions of P62 and LC3B-II were significantly upregulated (Figure 8F,G), indicating that METH-induced exosomes further impaired autophagic flux in SH-SY5Y cells.

**Scheme 1:**
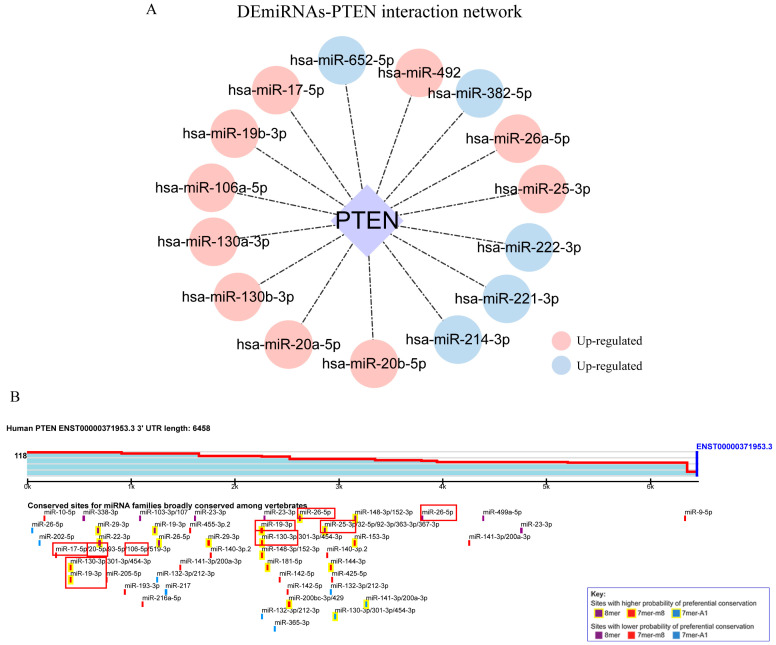
(**A**) The interaction network of DE-miRNAs and PTEN. (**B**) Prediction of PTEN-miRNA binding sites using TargetScan. The red rectangle highlights selected DE-miRNAs shown in (**A**).

We utilized TargetScan to predict miRNAs that interact with PTEN based on experimental evidence. The results indicated that miR-17-5p, miR-19b-3p, miR-106a-5p, and miR-25-3p may play important regulatory roles in PTEN within exosomes (Scheme 1A). The binding sites of these miRNAs to the PTEN gene are shown (Scheme 1B).

## 3. Discussion

The impact of METH on autophagy has been a focal point in investigating the neurotoxic mechanisms associated with METH. Xu et al. observed an upregulation of autophagy markers, including Beclin-1 and LC3-II, in the striatum of METH-exposed rats, leading them to conclude that METH promotes apoptosis through the induction of autophagy [2,5]. Similarly, Subu et al. proposed a correlation between the appearance of autophagy markers and apoptosis in the striatum of self-administered rats [10]. However, Huaisha et al. conducted a study in primary hippocampal neurons and suggested that inhibition of autophagy resulted in an increase in apoptotic markers [37]. The conflicting conclusions can be attributed to the dynamic nature of autophagy, which is challenging to fully comprehend solely based on changes at a specific time point or marker.

In our study, we present novel evidence elucidating the detrimental effects of METH on autophagy homeostasis, specifically its ability to impede neuronal autophagy flux. This disruption is characterized by an elevation in autophagosome synthesis and a concurrent inhibition of autophagosome transport and fusion, ultimately leading to impaired cytoplasmic metabolite degradation and neurotoxic damage. We also delved into the molecular mechanisms underlying these observations and reveal the pivotal role of Rab7a activation in modulating METH-induced autophagosome–lysosome fusion and subsequent metabolite degradation.

Our study confirmed the accumulation of autophagosomes induced by METH through both in vitro and in vivo experiments. To accurately assess the alterations in autophagy flux caused by METH, we utilized autophagy inhibitors to elucidate its effects on different stages of the autophagy process. Our findings revealed that METH increased the expression of autophagy markers in the presence of CQ and HCQ, indicating that METH promotes autophagosome synthesis, thereby contributing to their accumulation. This promotion of autophagy initiation by METH may be mediated through the ERK/mTOR pathway. Conversely, when the synthesis of new autophagosomes was blocked by 3-MA, METH still elevated autophagy markers, suggesting that METH also inhibits the degradation process of autophagosomes. This inhibition appears to result from impaired autophagosome transport and fusion with lysosomes, rather than a deficiency in lysosomal degradation capacity. Consequently, METH disrupts the delicate balance of autophagy flux, leading us to conclude that it exerts complex effects on autophagy flux, including both promotion of autophagosome synthesis and inhibition of degradation. The disruption of autophagy flux can result in the failure to degrade p-Tau protein, thereby eliciting neurotoxic effects.

As the sole member of the Ras-like GTPase superfamily specifically associated with lysosomes, Rab7a plays a critical role in regulating the transport process from late endosomes to lysosomes, thereby significantly influencing autophagy, particularly by facilitating the fusion between autophagosomes and lysosomes [11]. Our investigation unequivocally substantiated that METH disrupts orderly autophagic degradation by impeding Rab7a activity.

The functionality of Rab7a, a GTPase enzyme, is intricately regulated by its nucleotide binding state, which forms the basis for its diverse functions [38,39]. For instance, the inhibition of TBC1D5 can activate Rab7a, promoting the recruitment of reverse transcriptase to the nucleosome [33]. Additionally, vesicles carrying ATG9A contribute to the formation of mitochondrial autophagosomes by activating Rab7a, ensuring precise targeting near damaged mitochondria [16]. Endomorphic Igamma PIP5 kinase regulates endosomal maturation and lysosomal function by controlling Rab7a activity and localization [17]. Furthermore, LRRK1 inhibits autophagosome transport to lysosomes by inactivating Rab7a [36,40]. In these intricate processes, the activity status of Rab7a emerges as the pivotal determinant of its functionality, rather than alterations in its expression levels. In vitro studies have confirmed that depletion or dysfunction of Rab7a does not impede the initial fusion step of autophagosomes but leads to the accumulation of late autophagosomes and the loss of autolysosomes [15], preventing the maturation of autophagosomes into degrading autolysosomes.

In our investigation, we observed a notable decrease in PTEN levels and an increase in Rab7a phosphorylation following METH exposure. These findings suggest that METH may downregulate PTEN expression, thereby hindering the dephosphorylation of Rab7a and consequently reducing its activity. Previous studies have demonstrated that PTEN plays a crucial role in regulating the late endocytic pathway and EGFR degradation by dephosphorylating Rab7a [19]. However, further experimental validation is necessary to confirm this hypothesis.

In particular, our investigation revealed that exosomes released by METH-induced neurons promote neuronal autophagy. Our research findings indicate that the miRNA profiles in exosomes from METH-induced neurons significantly differ from those in the control group. Bioinformatics analysis reveals that the functions of these differentially expressed miRNAs (DE-miRNAs) are highly correlated with the autophagy process. Target-gene-miRNA interaction network analysis shows that PTEN interacts significantly with multiple important DE-miRNAs. Furthermore, we verified the impact of exosome treatment on the miRNA expression profile in SH-SY5Y cells and its effect on PTEN protein downregulation. Additional detection results indicate that these exosomes hinder autophagic flux by interfering with PTEN/Rab7a function.

Although there is no literature reporting the influence of exosomes on neuronal autophagy in current methamphetamine neurotoxicology studies, previous research has demonstrated the significant role of exosomes in regulating autophagy. For instance, Wenqiang Xin et al. reported that M2 anti-inflammatory microglial-derived exosomes reduce neuronal autophagy and apoptosis, thereby improving neurological function after stroke [41]. Min Guo et al. discovered that microglial exosomes facilitate α-synuclein transmission and impair autophagic flux in Parkinson’s disease [42]. Xue Zeng et al. found that exosomes in cerebral ischemia-reperfusion injury exacerbate the blockage of autophagosome degradation [43]. Hong-Xu Chen et al. demonstrated that exosomes derived from mesenchymal stem cells repair a Parkinson’s disease model by inducing autophagy [44]. These studies have revealed the significant roles of exosomes in the nervous system and neuroautophagy. Through the enrichment analysis of DEmiRNAs in exosomes, we found that these miRNAs not only participate in the PTEN/RAB7A pathway but also target multiple key pathways previously implicated in METH-induced autophagy, including the PI3K/AKT, mTOR, and ERK/MAPK signaling pathways, as well as genes such as Bcl2, DDIT4, and autophagy-related genes (ATGs). This suggests that exosomal miRNAs may regulate neuronal autophagy via a complex signaling network. Notably, exosomes possess the ability to protect their contents from degradation and exhibit excellent biocompatibility, making them promising candidates for future drug delivery systems aimed at treating METH-induced neuronal damage, with significant therapeutic potential. However, in the field of METH-induced neurotoxicity, the mechanisms by which exosomes exert their effects remain to be thoroughly investigated.

Limitations and Future Directions:

1. Limitations of the Cell Model: Studies conducted solely using the SH-SY5Y cell line in vitro may not comprehensively represent the effects of METH on diverse neuronal cell types. To validate the generalizability of the findings, it is advisable to incorporate additional cell lines, such as HT22 mouse hippocampal neurons or primary neuronal cultures, in parallel experiments.

2. Insufficient in vivo experimental intervention: The current in vivo studies investigating the expression of RAB7A and PTEN genes in mouse neurons lack sufficient depth. To further elucidate the role of the PTEN/Rab7a pathway in neuronal autophagy, it is recommended to employ gene editing techniques, such as CRISPR-Cas9, to specifically knockout or overexpress these genes. This approach would provide more definitive insights into their functional significance.

3. The mechanism of exosomal miRNA remains unclear: While it has been established that the altered expression profile of miRNAs in METH-induced neuronal exosomes influences autophagy, the specific miRNAs that play a predominant role remain unidentified. To elucidate their regulatory effects on the PTEN/Rab7a pathway and autophagy, it is essential to construct models for individual miRNA overexpression or knockdown. Furthermore, isolating neuron-specific extracellular vesicles in vivo presents significant challenges. Optimizing experimental methods (e.g., Proximity barcoding assay for single vesicle membrane proteomics) or combining these studies with complementary research approaches (e.g., Characteristic separation based on multiple exosomal markers of neurons) may help address this limitation.

4. Insufficient investigation of METH concentration and treatment duration: The effects of varying METH concentrations and treatment durations on autophagy have been only partially explored, with current research primarily limited to a concentration of 2.0 mM and a treatment duration of 24 h. To achieve a comprehensive understanding of the relationship between METH concentration, treatment time, and autophagy, it is essential to establish a broader range of concentration gradients and time points for systematic analysis.

5. Mechanism research lacks depth: The current study does not thoroughly investigate the mechanisms by which METH induces exosome release and miRNA sorting into exosomes. To address this question, it is recommended to employ proteomics and transcriptomics techniques to analyze changes in protein and gene expression profiles before and after METH treatment. This approach would help elucidate the underlying molecular mechanisms involved in these processes.

## 4. Materials and Methods

### 4.1. Animal Protocol

Fifty-four healthy adult male C57BL/6 mice (8w) were purchased from the Laboratory Animal Center of Southern Medical University (Guangzhou, China) and housed individually in tub cages in a temperature-controlled room with a 12 h light–12 h dark cycle and food and water available ad libitum. Mice were kept in these conditions for one week before treatment.

Mice were separated into 2 groups and subjected to the following treatment regimens: (1) mice administered saline injections were used as the control group; (2) mice administered METH (10 mg/kg) for 14 days via intraperitoneal (i.p.) injection at 12 h intervals were used as chronic METH group. The dose administered was based on our and other previous studies [45,46], and the exposure paradigm was chosen to mimic human METH abuse. In the establishment of the chronic METH poisoning model, both the saline group and the METH-treated group comprised 9 mice each.

Hydroxychloroquine sulfate (HCQ, Macklin, Shanghai, China), a validated lysosomotropic autophagy inhibitor which can easily cross the blood–brain barrier, was dissolved in saline. In accordance with the methodologies described by Mario Mauthe et al. [47], Quentin McAfee et al. [48], and Shipra Kartik et al. [49], HCQ (60 mg/kg) was administered intraperitoneally to mice once daily for 3 consecutive days, coinciding with the final 3 days of METH administration, to evaluate its effect on autophagic flux [47,49]. Specifically, HCQ treatment was initiated 1 h post-administration of either saline or METH. In the experiments for METH exposure and HCQ treatment, we randomly and evenly allocated the mice to four experimental groups, the saline control group, the METH treatment group, the HCQ treatment group, and the METH + HCQ treatment group, with each group containing nine mice.

After 12 h of treatment with vehicle saline, METH, or HCQ, the mice were euthanized under pentobarbital sodium anesthesia (200 mg/kg, i.p.) and sacrificed to obtain experimental samples.

All animal experiments were carried out according to the NIH Guidelines for the Care and Use of Laboratory Animals [50] and were approved by the Institutional Animal Care and Use Committee at the Southern Medical University (Ethical number: L2022125).

### 4.2. Cell Culture and Transfection

The human neuroblastoma cell line SH-SY5Y (CRL-2266, ATCC (American Type Culture Collection), Manassas, VA, USA) was bought from Pricella and cultured in a DMEM Nutrient Mixture F-12 (Ham) (Gibco, Grand Island, NE, USA) containing 10% fetal bovine serum (FBS; FSP500, ExCell Bio, Suzhou, China) and 1% antibiotics (Gibco, Grand Island, NE, USA). Cell cultures were maintained at 37 °C in a humidified atmosphere of 5% CO_2_.

The in vitro cell protocols are consistent with earlier studies from our lab [51], including the cell culture protocols and the design of drug treatment concentration and time. SH-SY5Y cells were initially exposed to METH concentrations of 0, 1.0 mM, 2.0 mM, and 3.0 mM for 24 h, and exposed to 2.0 mM METH for 0 h, 12 h, 24 h, and 36 h.

As inhibitors of autophagy, chloroquine (CQ, 200 nM, C843545, Macklin, Shanghai, China) and 3-Methyladenine (3-MA, 5.0 mM, HY-19312, MedChemExpress, Shanghai, China) were dissolved in dimethyl sulfoxide (DMSO, D8371, Solarbio, Beijing, China) and added to the medium 1 h prior to METH treatment. The final concentration of DMSO in the culture medium did not exceed 0.1% (*v*/*v*). As an autophagy inducer, rapamycin (Rapa, 500 nM, HY-10219, MedChemExpress, Shanghai, China) was dissolved in saline and similarly added to the medium 1 h before METH treatment. In the control group, equal volumes of the corresponding solvents (normal saline or DMSO) from the same batch were used.

Plasmid constructs: The full-length coding sequence of the human RAB7A gene (NM_004637.6) was amplified from HEK293T cDNA and subsequently cloned and inserted into CS2-Myc-GFP vectors using XhoI and EcoRI restriction endonucleases (New England Biolabs, Ipswich, MA, USA) with double digestion. The primers used to identify the product of the connection between the CS2-Myc vector and the RAB7A fragment are in Table 1. The T22N and Q67L mutations were introduced into the wild-type CS2-Myc-RAB7A plasmids via PCR-based site-directed mutagenesis. Recombinant plasmids were purified using the Endo Free Mini Plasmid Kit (4992422, TIANGEN BIOTECH, Beijing, China) and validated using Sanger sequencing.

Cell Transfection and Treatment: SH-SY5Y cells were seeded in 6-well plates and cultured until they reached 70% to 80% confluence. Transfection was then performed using the Lipofectamine 3000 reagent and the corresponding plasmids (CS2-Myc-GFP, CS2-Myc-RAB7A T22N, or CS2-Myc-RAB7A Q67L). After gentle mixing, the cells were incubated at 37 °C with 5% CO_2_ for 4–6 h. The medium was subsequently replaced with complete growth medium, and the cells were further cultured for an additional 24 h. Following this, the transfected cells were re-seeded into new 6-well plates. When the cell confluence reached 80%, the cells were treated with either saline or METH.

### 4.3. Immunofluorescence Labelling

The mice were euthanized for sample collection 12 h following the final METH injection. The brain and other tissues were rapidly subjected to fixation or cryopreservation treatment. The brain tissue was dehydrated with 30% sucrose and embedded with optimal cutting temperature compound. Then, coronal sections were prepared on a cryostat (ZEISS, Oberkochen, Germany) and the slices (40 μm) from the striatum region were selected for subsequent experiments.

After being permeabilized for 10 min with 10% Triton X-100 (diluted with PBS), frozen brain (striatum) slices were blocked for 1 h at room temperature with 10% goat serum. Then the slices were incubated with the primary antibody for a whole night at 4 °C. With the appropriate dose of fluorescently labelled secondary antibody, the sections were incubated at room temperature for 1 h in darkness.

SH-SY5Y cells were plated in a confocal dish, fixed in 4% paraformaldehyde, permeated with 0.1% Triton X-100 for 5 min, and then blocked with 10% goat serum. The following steps were similar to the above.

The primary antibodies were as follows: LC3B (1:500, ab192890, Abcam, Shanghai, China); SQSTM1/p62 (1:500, ab109012, Abcam, Shanghai, China); Rab7a (1:500, ab137029, Abcam, Shanghai, China); RILP antibody (1:500, #25472, Signalway Antibody, Greenbelt, MA, USA); LAMP1 (1: 100, 65051-1-IG-100UG, Proteintech, Wuhan, China). Microphotographs were collected using a fluorescence microscopy (FV3000, Olympus, Tokyo, Japan). All digital images were processed to improve the contrast using the same settings only by adjusting the contrast and brightness in the OLYMPUS FV31S-SW software (Version: 2.4.1.198, Olympus, Tokyo, Japan). The qualitative analyses of the area of positive pixels, fluorescence intensity, and co-localization areas of fluorescence pixels across different channels in the images were conducted using the Fiji-imageJ (Version: 1.54f, Wayne Rasband and contributors, National Institutes of Health, Bethesda, MA, USA).

### 4.4. Electron Microscopy

SH-SY5Y cells were fixed with ice-cold 2.5% glutaraldehyde (Sigma-Aldrich, St. Louis, MO, USA) at 4 °C for 1 h. Then, the cells were sent to the Central laboratory of Southern Medical University for ultra-thin sectioning (0.1 mm thin section). Briefly, after being fixed with 2.5% glutaraldehyde and 1% osmium tetroxide, the samples were dehydrated with graded ethanol and impregnated with graded resin before being embedded in embedding molds. They were then subjected to ultrathin sectioning. The sections were stained with 2% uranyl acetate saturated aqueous solution and lead citrate for electron microscopy observation. Images were taken using an H-7500 electron microscope (Hitachi, Chiyoda, Japan) at 60KV.

### 4.5. Lysosomal Acidity Analysis

LysoSensor™ Yellow/Blue DND-160 (PDMPO, 40768ES, Yeasen Biotechnology, Shanghai, China) was utilized for lysosomal fluorescent staining. SH-SY5Y cells were seeded in confocal dishes and cultured until they reached approximately 70% confluence. The cells were then exposed to METH for 24 h, after which the culture medium was replaced. Subsequently, the cells were incubated with a 4 μM of a PDMPO solution for 30 min at 37 °C in the dark, followed by three washes with PBS. Live cell imaging was acquired using fluorescence microscopy (FV3000, Olympus, Tokyo, Japan). Lysosomes were observed using dual-channel excitation/emission wavelengths (Ex/Em = 329 nm/440 nm and Ex/Em = 384 nm/540 nm). LysoSensor exhibits pH-dependent enhancement of fluorescence intensity, with increased fluorescence as the degree of organelle acidification increases. In a neutral environment, these probes emit blue fluorescence (Ex/Em = 329 nm/440 nm), while in an acidic environment, they emit yellow fluorescence (Ex/Em = 384 nm/540 nm).

### 4.6. Western Blotting

The protocol used for the analysis of brain tissues and cell proteins using the standard operating procedure for a Western blotting protocol. The primary antibodies were as follows: LC3B (1:1000, ab192890, Abcam, Shanghai, China); SQSTM1/p62 (1:1000, ab109012, Abcam, Shanghai, China); Rab7a (1:1000, 55469-1-AP, Proteintech, Wuhan, China); p-RAB7a (phosphoS72) (1:1000, ab302494, Abcam, Shanghai, China); RILP (1:500, #25472, Signalway Antibody, Greenbelt, USA); GAPDH (1:1000, 60004-1-IG, Proteintech, Wuhan, China); PTEN (1:1000, 9559, Cell Signaling Technology, Shanghai, China); p-Tau (T181) (1:1000, ab254409, Abcam, Shanghai, China); p-mTOR (Ser2448) (1:2000, 67778-1-Ig, Proteintech, Wuhan, China); p-ERK1/2 (Thr202/Tyr204) (1:1000, 28733-1-AP, Proteintech, Wuhan, China); LAMP1 (1: 1000, 65051-1-IG-100UG, Proteintech, Wuhan, China). The secondary antibodies were 115-035-003 (Goat anti rabbit) and 111-035-003 (Goat anti mouse) (1:10,000, Jackson ImmunoResearch, West Grove, PA, USA). The immunoblot bands were visualized using an ECL kit (Focus, Shanghai, China) and acquired on Tanon 5200 system (Tanon, Shanghai, China). For each protein of interest, we repeated three independent experiments and selected the representative band, as shown in this manuscript. Data of related protein levels were obtained from the bands calculated using Fiji-ImageJ (Version: 1.54f, Wayne Rasband and contributors, National Institutes of Health, Bethesda, MD, USA).

### 4.7. Co-Immunoprecipitation

SH-SY5Y cells were cultured in 10 cm diameter tissue culture dishes and maintained at 37 °C in a 5% CO_2_ atmosphere. After 48 h, the cells were treated with 2.0 mM METH for 24 h. Subsequently, the cells were lysed using cell lysis buffer (P0013, Beyotime Biotechnology, Shanghai, China) supplemented with 1.0 mM phenyl methane sulfonyl fluoride (PMSF, Beyotime Biotechnology, Shanghai, China) for 30 min. Cellular debris was removed by centrifugation at 12,000× *g* for 10 min. The resulting supernatants were incubated with appropriate amounts of RAB7A antibody (1:50, sc-376362, Santa Cruz Biotechnology, Shanghai, China) and control rabbit IgG (1:100, SC-2027, Santa Cruz, Shanghai, China) and enriched using Protein A/G PLUS-Agarose (sc-2003, Santa Cruz, Shanghai, China) for 6 h at 4 °C. Non-specific binding was eliminated by washing the beads three times with PBS. The bead-bound proteins were then eluted by incubating with a 1× protein loading buffer at 95 °C for 10 min for subsequent SDS-PAGE analysis to detect the target protein.

### 4.8. Extraction and Application of Exosomes

The extraction of exosomes is performed by means of size exclusion chromatography (SEC) and ultracentrifugation (UC). In brief, exosome-free fetal bovine serum was prepared by ultracentrifugation at 100,000× *g* for 18 h [52]. When the SH-SY5Y cells had expanded to a certain scale, the serum in the culture medium was substituted with exosome-free serum, and the cells were treated with PBS or 2.0 mM METH. The cell culture supernatant (150 mL/group) was collected after treatment for 48 h, and then it was centrifuged at 2000× *g* for 10 min and 10,000× *g* for 45 min at 4 °C. After that, the supernatants without contamination by cellular debris were collected for ultrafiltration concentration (Amicon^®^ Ultra-15mL 100K, UFC910096, Millipore, Burlington, VT, USA). The concentrated supernatant was separated using Izon’s qEV separation column (qEVoriginal-35 nm, Izon, Christchurch, New Zealand) according to the instructions for exosome isolation. The exosomes collected were purified by ultracentrifugation at 100,000× *g* for 70 min at 4 °C.

The exosomes obtained were identified by exosome particle detection-nanoparticle tracking analysis (NTA), transmission electron microscopy (TEM), and Western blot analysis of marker proteins, or used directly in experiments after being quantified by BCA protein assay or stored in PBS solution containing trehalose at −80 °C. In experiments, exosomes were added to the cell culture medium at a concentration of 10 μg/mL (exosome protein content/cell culture volume) for treatment.

### 4.9. Identification of Exosomes

(1) NTA: NTA was utilized to quantify and statistically analyze the size distribution of exosomes. Exosome samples are diluted with 1 × PBS and used directly for NTA detection (Instrument Model: ZetaVIEW S/N 17-310, analysis Software Version: ZetaView 8.04.02, Particle Metrix GmbH, Ingolstadt, Germany).

(2) TEM: TEM was employed to verify whether the morphological characteristics of the isolated sample conform to those of exosomes. A total of 5 μL of the exosome sample was dropped onto a copper grid and incubated at room temperature for 5 min. After the incubation is completed, the excess liquid was blotted from one side with blotting paper. A drop of 2% uranyl acetate was added to the copper grid and incubated at room temperature for 1 min. After the incubation was completed, the excess liquid was blotted from one side with blotting paper. It was dried at room temperature for 20 min, and then detected using a nano transmission electron microscope (Tecnai G2 Spirit BioTwin, Thermo Fisher Scientific, Shanghai, China) with the accelerating voltage set at 80 kV.

(3) Western blot analysis of marker proteins: The dissociation buffer containing 2% SDS was used to disrupt exosomes. The general steps are as described in the Western blotting section with CD63 (1:1000, ab315108, Abcam, Shanghai, China), CD81 (1:5000, ab109201, Abcam, Shanghai, China), and TSG101 (1:5000, ab125011, Abcam, Shanghai, China).

### 4.10. MiRNA Array Analysis

Exosome miRNA extraction was performed according to the instructions provided in the mirPremier^®^ miRNA isolation kit (SNC50, Merck Millipore, Shanghai, China). The extracted miRNA was loaded onto an miRNA array (Affy miRNA array, H·Wayen Biopharmaceutical Technology Co., Ltd., Shanghai, China) for detection and analysis.

### 4.11. Prediction of Differentially Expressed miRNA Target Genes and Bioinformatics Analysis

The mirtarBase database [53] and Targetscan database (https://www.targetscan.org/vert_80/, accessed on 25 February 2025) were employed to predict the target genes of differential miRNAs. Gene Ontology (GO, http://geneontology.org/, accessed on 11 December 2023) and Kyoto Encyclopedia of Genes and Genomes (KEGG, https://www.genome.jp/kegg/, accessed on 11 December 2023) pathways analysis were conducted using ClusterProfiler (v4.8.3, T. Wu et al.) [54] and the R project (v4.3.1, R Core Team, 2023 [55]), and RStudio version: 2023.12.0+369 “Ocean Storm” Release, Ushey K et al., 2023 [56,57]). Cytoscape (v3.10.1) [58] was employed to create a regulatory network diagram of miRNA and target genes in cellular connections.

### 4.12. Quantitative Real-Time PCR Validation of miRNA Expression

RNA extraction was performed using Trizol reagent (15596026, Invitrogen, Shanghai, China) following the manufacturer’s standard operating procedures. miRNA-specific forward primers (Table 2) were designed based on the complete miRNA sequences retrieved from the miRBase database (https://www.mirbase.org/, accessed on 3 March 2021). miRNA reverse transcription was conducted using the miRNA 1st Strand cDNA Synthesis Kit (by tailing A) (MR201, Vazyme, Nanjing, China), according to the manufacturer’s protocol. The procedure involved mixing the total RNA template, enzymes, and miRNA RT Mix, followed by incubation at 37 °C for 60 min and then at 85 °C for 5 min to obtain the cDNA.

Quantitative Real-Time PCR (qPCR) was conducted using the miRNA Unimodal SYBR qPCR Master Mix reagent kit (Product No.: MQ102, Vazyme). The 20 μL reaction mixture was prepared according to the manufacturer’s instructions. For the miRNA tailing method, the Universal Reverse Q Primer provided in the kit served as the universal reverse primer. U6 small nuclear RNA was selected as the internal reference gene. The qPCR amplification protocol consisted of an initial denaturation at 95 °C for 30 s, followed by 40 cycles of denaturation at 95 °C for 5 s and annealing/extension at 60 °C for 30 s. Melt curve analysis was subsequently performed from 65 °C to 95 °C with 0.5 °C increments every 5 s. The relative expression levels of miRNA and U6 were calculated using the 2^−(ΔΔCt)^ method (Livak method).

### 4.13. Statistical Analysis

Statistical data are presented as mean ± standard error of the mean (SEM). The statistical tests selected were parametric tests or non-parametric tests according to normality tests and homogeneity tests of variance. When the data met the normal distribution and homoscedasticity, a *t*-test or ANOVA test was used, and when the variances were not equal, methods of correction by test were used. When the data did not meet the normal distribution, a Mann–Whitney test or other non-parametric statistical methods were used; multiple comparisons used the corrected post-test method. A *p* value < 0.05 was considered statistically significant and all statistical tests were two-tailed. Statistical tests were performed using the software Prism (v8.0.2, GraphPad Software Inc., San Diego, CA, USA).

## 5. Conclusions

Our findings indicate that METH induces autophagosome accumulation in neurons, which results from both increased autophagosome production and inhibited autophagosome degradation. Specifically, METH inhibits autophagosome degradation by suppressing Rab7a activity through PTEN-mediated dephosphorylation regulation. Additionally, METH-induced changes in the exosomal miRNA profiles of neurons can modulate Rab7a and consequently affect autophagy by altering PTEN expression levels.

In conclusion, the impact of METH on neuronal autophagy primarily involves its regulation of Rab7a activity, which significantly impairs the transport and fusion of autophagosomes. Additionally, METH-induced neuronal exosomes further exacerbate this process through miRNA-mediated mechanisms. This pioneering study not only provides novel insights and research directions for the field of METH-induced neurotoxicity but also identifies a promising target for the prevention and treatment of METH-related toxic damage.

## Figures and Tables

**Table 1:** Primers utilized for the identification of the product resulting from the ligation between the CS2-Myc vector and the RAB7A gene fragment.

Primer Name	Primer Sequence (5′-3′)	Primer Length
Ecor1-RAB7-F	CCGGAATTCATGACCTCTAGGAAGAAAGTG-	30 bp
Xhol-RAB7-R	CCGCTCGAGTCAGCAACTGCAGCTTTCTG-	29 bp

**Table 2:** Primers used for qPCR validation of miRNA expression in exosomes and exosome-treated SH-SY5Y cells.

Primer Name	Primer Sequence (5′-3′)	Primer Length
hsa-miR-15b-5p-F	TAGCAGCACATCATGGTTTACA	22 bp
hsa-miR-16-5p-F	TAGCAGCACGTAAATATTGGCG	22 bp
hsa-miR-17-5p-F	CAAAGTGCTTACAGTGCAGGTAG	23 bp
hsa-miR-19b-3p-F	TGTGCAAATCCATGCAAAACTGA	23 bp
hsa-miR-106a-5p-F	AAAAGTGCTTACAGTGCAGGTAG	23 bp
hsa-miR-503-5p-F	TAGCAGCGGGAACAGTTCTGCAG	23 bp
hsa-miR-25-3p-F	AACCTCCCATTGCACTTGTCTC	22 bp

## Data Availability

The data and materials of the study are available upon request from the corresponding author.
